# Alpine viper in changing climate: thermal ecology and prospects of a cold-adapted reptile in the warming Mediterranean

**DOI:** 10.1038/s41598-024-69378-4

**Published:** 2024-08-16

**Authors:** Edvárd Mizsei, Dávid Radovics, Gergő Rák, Mátyás Budai, Barnabás Bancsik, Márton Szabolcs, Tibor Sos, Szabolcs Lengyel

**Affiliations:** 1 Conservation Ecology Research Group, Institute of Aquatic Ecology, HUN-REN Centre for Ecological Research, Budapest, Hungary; 2 Kiskunság National Park Directorate, Kecskemét, Hungary; 3 Department of Ecology, University of Debrecen, Debrecen, Hungary; 4 Department of Systematic Zoology and Ecology, Eötvös Loránd University, Budapest, Hungary; 5 Department of Ecology, University of Veterinary Medicine, Budapest, Hungary; 6 Evolutionary Ecology Group, Hungarian Department of Biology and Ecology, Babeş-Bolyai University, Cluj-Napoca, Romania; 7 Milvus Group Bird and Nature Protection Association, Tîrgu Mureş, Romania; 8 Biodiversity, Climate Change and Water Management Coordination Research Centre, University of Debrecen, Debrecen, Hungary; 9 Institute of Metagenomics, University of Debrecen, Debrecen, Hungary

**Keywords:** Thermoregulation, Thermal behaviour, Climate change, Ecological niche, Population-level variation, Time budget, *Vipera graeca*, *Vipera ursinii*, Herpetology, Climate-change ecology, Conservation biology, Ecological modelling

## Abstract

In a rapidly changing thermal environment, reptiles are primarily dependent on in situ adaptation because of their limited ability to disperse and the restricted opportunity to shift their ranges. However, the rapid pace of climate change may surpass these adaptation capabilities or elevate energy expenditures. Therefore, understanding the variability in thermal traits at both individual and population scales is crucial, offering insights into reptiles' vulnerability to climate change. We studied the thermal ecology of the endangered Greek meadow viper (*Vipera* *graeca*), an endemic venomous snake of fragmented alpine-subalpine meadows above 1600 m of the Pindos mountain range in Greece and Albania, to assess its susceptibility to anticipated changes in the alpine thermal environment. We measured preferred body temperature in artificial thermal gradient, field body temperatures of 74 individuals in five populations encompassing the entire geographic range of the species, and collected data on the available of temperatures for thermoregulation. We found that the preferred body temperature (*T*_*p*_) differed only between the northernmost and the southernmost populations and increased with female body size but did not depend on sex or the gravidity status of females. *T*_*p*_ increased with latitude but was unaffected by the phylogenetic position of the populations. We also found high accuracy of thermoregulation in *V.* *graeca* populations and variation in the thermal quality of habitats throughout the range. The overall effectiveness of thermoregulation was high, indicating that *V.* *graeca* successfully achieves its target temperatures and exploits the thermal landscape. Current climatic conditions limit the activity period by an estimated 1278 h per year, which is expected to increase considerably under future climate scenarios. Restricted time available for thermoregulation, foraging and reproduction will represent a serious threat to the fitness of individuals and the persistence of populations in addition to habitat loss due to mining, tourism or skiing and habitat degradation due to overgrazing in the shrinking mountaintop habitats of *V.* *graeca*.

## Introduction

Reptiles are exposed to increasing threats worldwide, with about 20% of species facing the risk of extinction due to the interaction or synergy of factors including habitat loss, fragmentation and degradation, environmental pollution and spread of diseases, among others^1,2^. These impacts are further intensified by the rapidly changing thermal environment, which present significant ecological and physiological challenges to all ectotherms^3^. Despite their widely acknowledged thermal constraints, increasing evidence underscores the diverse coping strategies that reptiles employ to adapt to variation in climate. These strategies may include physiological and behavioral plasticity, shifting their geographic ranges, or ultimately undergoing evolutionary adaptation^4–6^. Central to these responses is the essential role of thermal trait variation. The evolution of all physiological and behavioral traits hinges on the existence of inherent phenotypic and genetic variation, which is presumed to be linked to fitness^7^. Therefore, comprehending the variability in thermal traits at the individual and population level is essential. This understanding can serve as a predictive tool for evaluating reptiles' vulnerability to climate change^8,9^.

Temperature is a critical environmental factor which determines the geographical distribution and abundance of populations of any species, particularly reptiles^10,11^. Ectotherms depend on effective thermoregulation, balancing physiological and behavioural mechanisms to maintain their body temperature (*T*_*b*_) within an optimal range^12^. This temperature regulation is crucial for essential life processes, including foraging, reproduction, and predator avoidance^13^. Reptiles exhibit a range of thermoregulatory strategies, influenced by the specific environmental conditions and the relative costs and benefits of various thermoregulatory behaviours^14,15^. These strategies span from thermoconformity, where there is minimal active temperature regulation, to active thermoregulation, where reptiles actively seek or avoid heat sources to control their body temperature^16^. The variability in thermoregulatory behaviours among reptile species reflects their adaptation to local environmental conditions^17,18^. For instance, the timing of daily and seasonal activities plays a significant role in determining body temperature, furthermore reptiles often select specific thermal microhabitats that facilitate optimal thermoregulation during active periods^5^. The rapid changes in temperature, particularly warming, are pushing many species towards the limits of their thermal tolerance^19^. This shift leads to altered activity patterns, which can result in reduced fitness, and ultimately in changes in population size and distribution^3^. Cold-adapted species and those in higher latitudes or altitudes are, particularly, exposed to acute challenges due to significant temperature increases, as their narrow thermal tolerances could amplify their vulnerability^20^.

Forecasting the impacts of environmental change on reptiles depends on evaluating reptiles' ability to regulate their *T*_*b*_^21^. However, even differentiating whether variations in *T*_*b*_ arise from environmental fluctuations (i.e., thermal constraints) or from changes in thermal behaviour or physiology, such as plasticity in preferred or optimal *T*_*b*_, represents a challenge. Fortunately, a detailed protocol for defining the accuracy and effectiveness of thermoregulation has been developed and refined over time^16,22,23^. Furthermore, the suite of thermal ecology traits defined as measurable proxies of thermoregulatory behaviors (Table 1)^21,24^, directly or indirectly influence an individual's habitat choice, temporal and spatial activity patterns, and behavior within the thermal environment^25–28^. Consequently, these traits also shape and can serve as indicators of the distribution and abundance patterns of populations or species within ecosystems^29,30^.

**Table 1:** Variables used in this study to assess the thermal ecology of *Vipera* *graeca*.

Index	Definition and interpretation
*T*_*e*_	Operative temperature is the *T*_*b*_ that a non-thermoregulating animal could attain based on radiation, conduction, and convection
*T*_*b*_	The internally measured temperature of a free-living animal's body
*T*_*set*_	The set-point range is the preferred body temperature, measured by the bounds of the central 50% of the distribution of body temperatures selected in a thermal gradient
*T*_*p*_	Peak value of the distribution of body temperatures selected in a thermal gradient
*d*_*b*_	Accuracy of body temperature of Hertz et al.^16^, measured as the mean of the deviations of *T*_*b*_ from *T*_*set*_. High values represent low accuracy because *T*_*b*_ is much higher or lower than preferred; values approaching 0 represent high accuracy
*d*_*e*_	Thermal quality of the habitat of Hertz et al.^16^, measured as the mean of the deviations of T_e_ from T_set_. High values represent low quality because *T*_*e*_ is much higher or lower than preferred; values approaching 0 represent high quality
*E*	Effectiveness of thermoregulation of Hertz et al.^16^, *E* = 1* − *(*d*_*b*_/*d*_*e*_). *E* will approach 0 when animals do not thermoregulate, will approach 1 when animals thermoregulate carefully, and values are negative when animals actively avoid thermoregulation
*I*	Effectiveness of thermoregulation of Boulin-Demers and Weatherhead^22^, *I* = *d*_*e*_* − d*_*b*_. *I* is 0 when animals thermoconform, is negative when animals avoid thermally favourable habitats and is positive when animals are thermoregulating. Higher values mean animals are better thermoregulators
*E*_*x*_	Thermal exploitation of the habitat of Christian and Weavers ^23^, calculated as the time in which the *T*_*b*_ of the animals were within *T*_*set*_, divided by the time which *T*_*e*_ was within *T*_*set*_. Higher values mean animals are better thermoregulators
*h*_*r*_	The number of hours *h*_*r*_/year that *T*_*e*_ exceeds *T*_*set*_. High values of *h*_*r*_ are associated with an increased risk of local extinction

In a rapidly changing thermal environment, local adaptation could be essential for the survival of reptile populations, given their limited ability to disperse and the narrow window of time available for shifting their distribution range or undergoing adaptive evolutionary changes^5,6,31^. Physiological and behavioral plasticity can help ectotherms cope with warming by reducing the thermal sensitivity of vital processes and enhancing physiological tolerances^32,33^. Additionally, these plastic responses can minimize exposure to harmful or lethal temperatures^11,34^. However, the pace of climate warming might outstrip the capacity for plastic responses or increase the costs associated with behavioral strategies^26,35,36^.

This study aims to comprehensively assess the thermoregulatory behaviour of *Vipera* *graeca*, a cold adapted endangered alpine snake particularly threatened by climate change and to evaluate its degree of vulnerability to its effects. Perviously, the thermoregulation of the species was almost unknown. In a recent paper, we suggested that vipers do not fully exploit the thermally optimal time window available to them, likely because they shift their activity to periods with fewer avian predators^13^, and we provided the measurement of voluntary thermal maxima (VTM), which revealed high upper thermal tolerance of grassland vipers in alpine environments, which was 36.6 °C in the case of *V.* *graeca*^37^.

We use data from on-site measurements, field tests and the thermal landscape to address five pivotal questions. (Q1) Does their preferred body temperature depend on the sex and body size of individuals? (Q2) Do the populations of *V.* *graeca* differ in preferred body temperature? (Q3) Are the differences in thermal characteristics among populations, if any, influenced by latitudinal or phylogenetic position? (Q4) To what extent can individuals accurately and effectively thermoregulate to achieve the preferred range of body temperatures and exploit the thermal landscape or quality of their habitats? (Q5) Do current climatic conditions represent thermal limitations for the activity of *V.* *graeca* and, if so, how will these limitations change concerning future climate change? By addressing these questions, the study aims to provide a detailed understanding of the thermoregulatory strategies and adaptations in *V.* *graeca*, considering a range of biological and environmental influences at both the individual and the population levels. Additionally, mitigating the potential impacts of climate change on these vipers is crucial for effective conservation efforts^38,39^.

## Results

**Figure 1:**
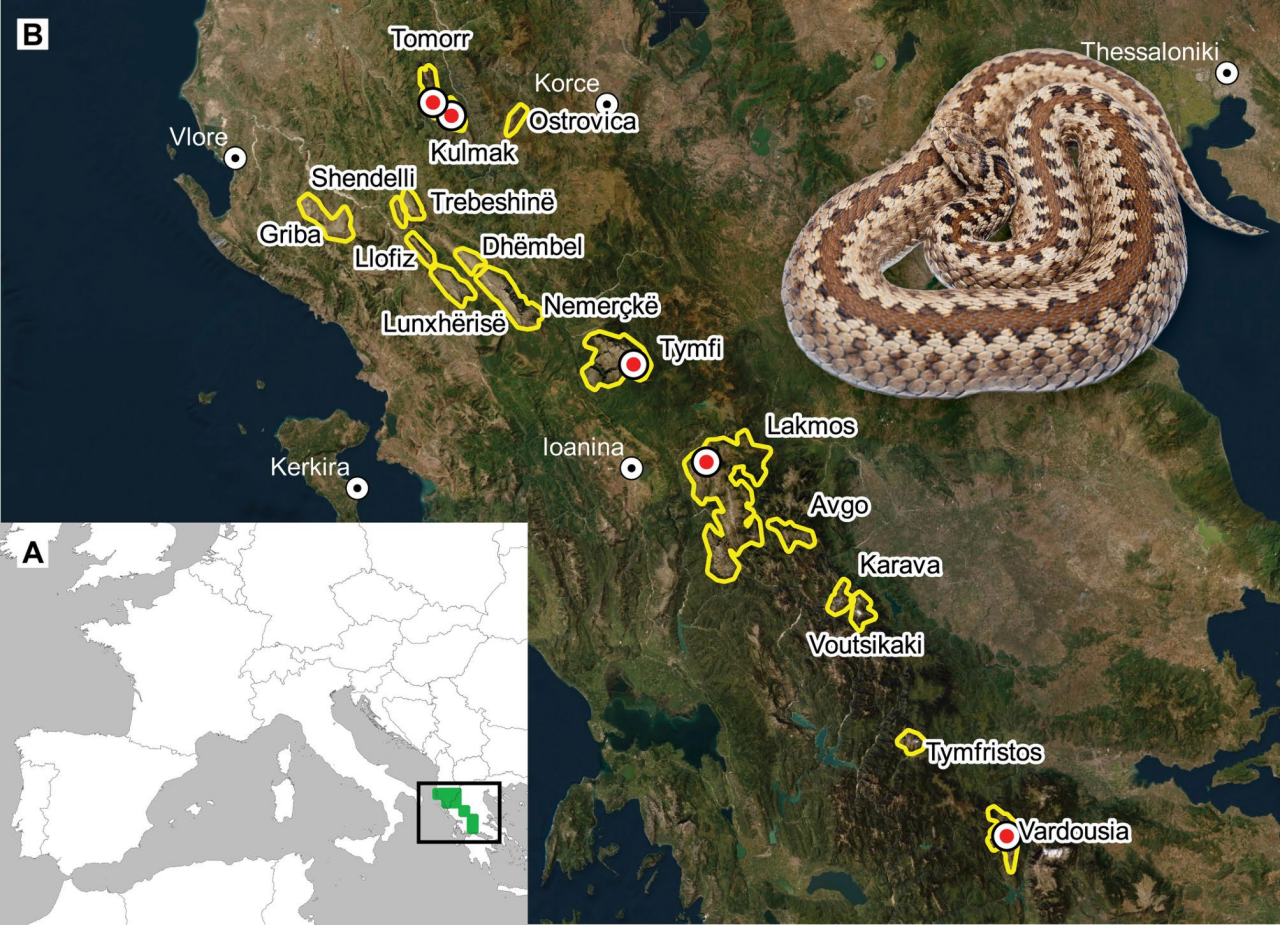
Panel (**A**) The distribution of *Vipera* *graeca* at 50 × 50 km spatial resolution Mizsei et al.^52^, and the area of panel (**B**) (black rectangle). Panel (**B**) Buffered polygons of suitable habitats of *V.* *graeca* based on a distribution model Mizsei et al.^46^ and sampling sites of this study (red dots), and a picture of a representative female *V.* *graeca* individual sampled during the data collection. Maps were generated in QGIS 3.14 using the QuickMapServices plugin (QGIS.org (2024). QGIS Geographic Information System. Open Source Geospatial Foundation Project. http://qgis.org).

**Figure 2:**
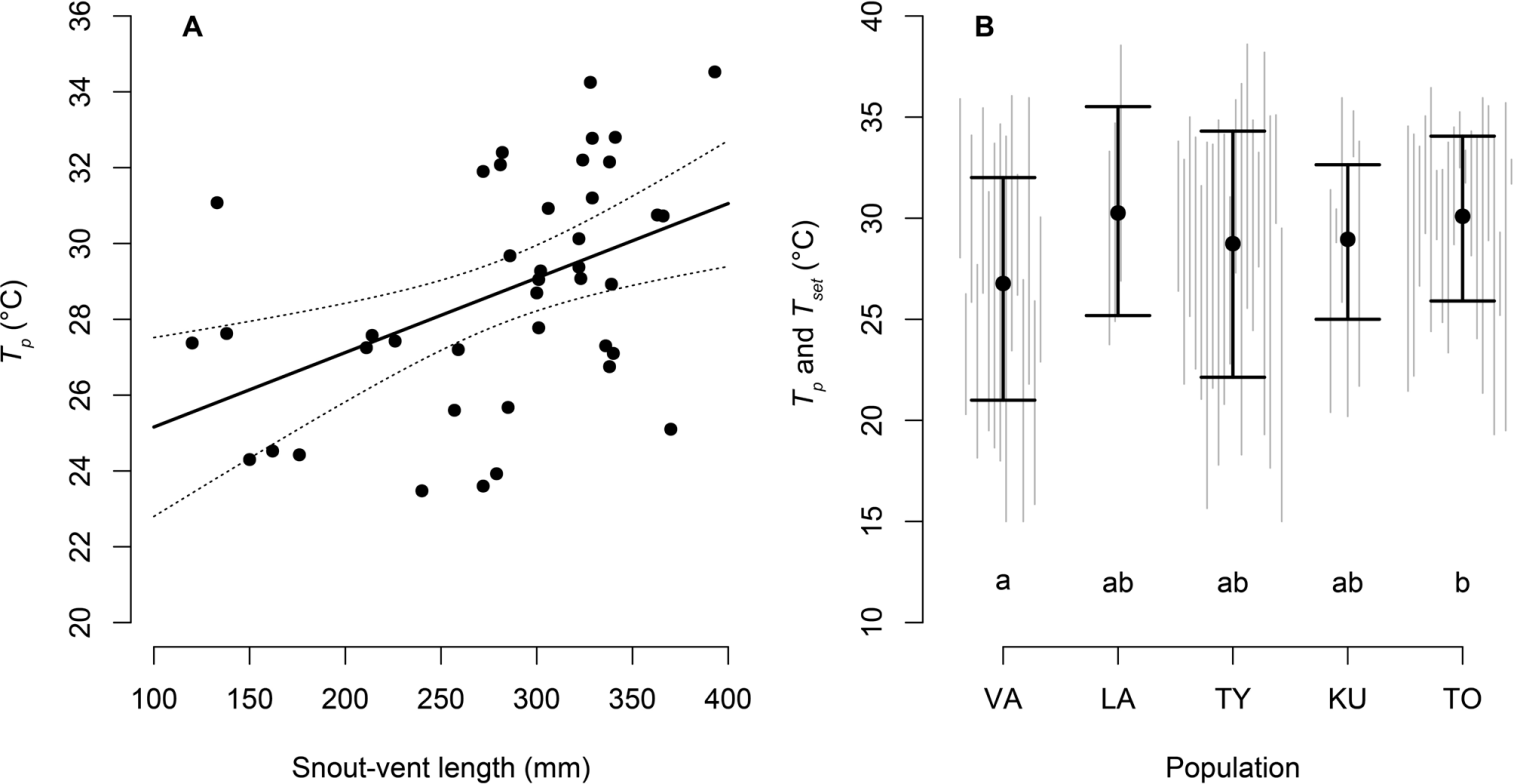
Influence of snout-vent length (SVL) on *T*_*p*_ in *Vipera* *graeca* females. Solid lines show the prediction of LM, and dashed lines delimit 95% CI (panel **A**). *T*_*set*_ (solid interval lines) and *T*_*p*_ (dots) of measured populations of *Vipera* *graeca* (panel **B**). Grey lines show individual *T*_*set*_ values. For populations acronyms see Fig. 1.

In total, we collected temperature data on n = 74 *Vipera* *graeca* individuals from five populations (Fig. 1; Table 2). The overall preferred body temperature (*T*_*p*_) was 28.77 ± 0.43 °C (mean ± SE, Table 2). The sex of individuals did not have an effect on *T*_*p*_ (Wilcoxon W = 425, *P* = 0.824; Linear model coefficient slope b = 0.146, *P* = 0.873). We found that in males, snout-vent length (SVL) did not influence *T*_*p*_ (b = − 0.009, *P* = 0.717), however, it had a significant positive effect in females (b = 0.02, *P* = 0.003; Fig. 2A). Gravid reproductive state in adult females did not influence *T*_*p*_ (W = 46, *P* = 0.23; b = 1.63, *P* = 0.22).

**Table 2:** Sample size of temperature measurements, number of individuals and snout-vent length (SVL) means recorded in this study.

Population	No of *T*_*e*_	No of *T*_*b*_	No of *T*_*set*_	No of individuals for *Tp* and *T*_*set*_	No of male individuals for *Tp* and *T*_*set*_	No of female individuals for *Tp* and *T*_*set*_	Mean of SVL (mm)	SE of SVL (mm)
TO	18,556	14	203	19	4	15	293.21	16.14
KU	18,892	5	34	6	1	5	234.33	40.3
TY	43,418	28	250	31	13	18	257.71	9.27
LA	17,579	3	67	3	–	3	317.67	39.57
VA	31,031		260	15	6	9	262.6	9.51

See Fig. 1. for the location of Population acronyms.

**Figure 3:**
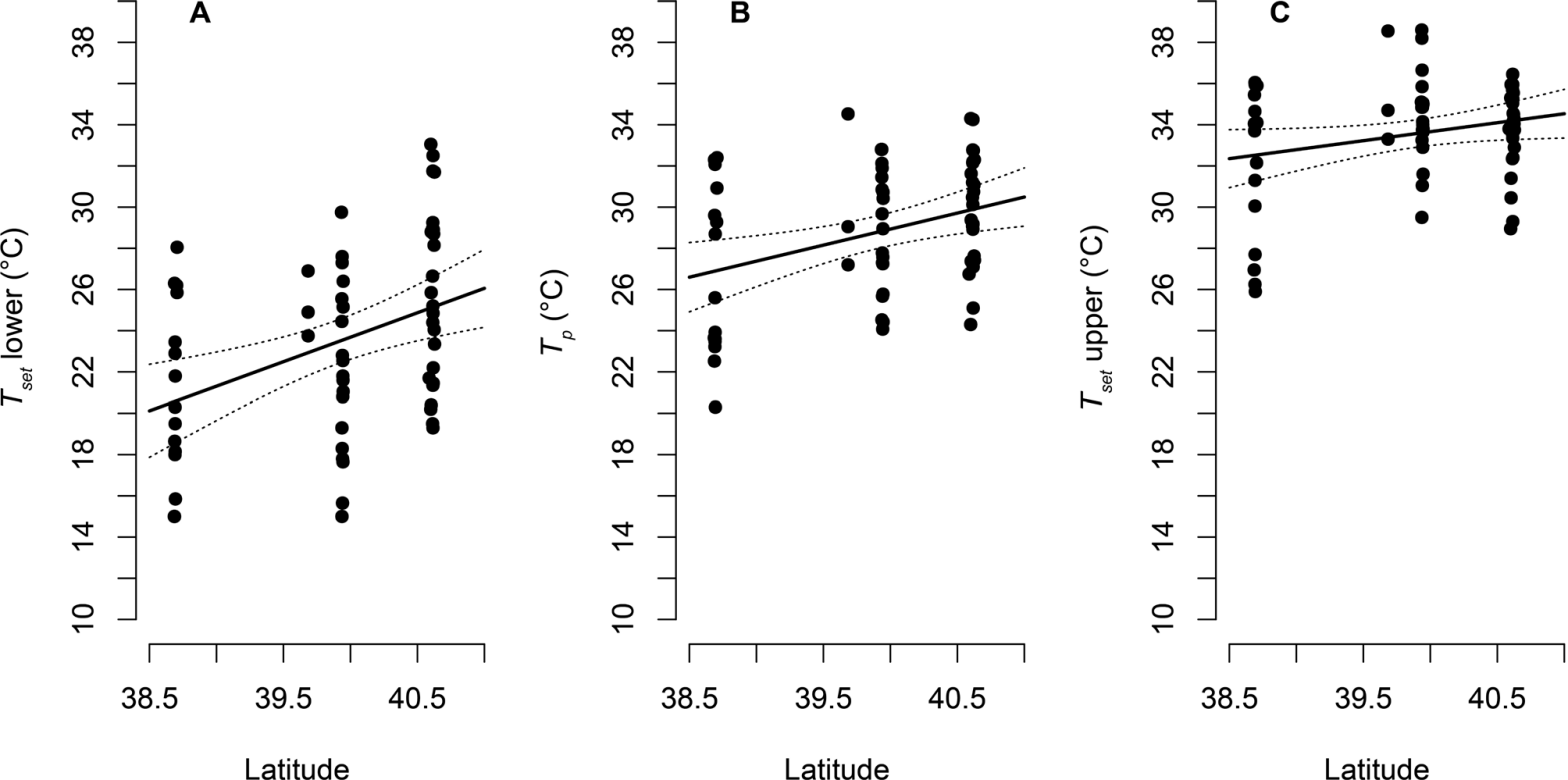
Influence of latitude on *T*_*set*_ and *T*_*p*_ of *Vipera* *graeca*. Dots show individual values, solid lines show prediction of LM, and dashed lines delimit 95% CI.

We found that most populations did not differ from each other in *T*_*p*_, except for a significant difference in *T*_*p*_ among the southernmost and northernmost localities, the Vardoussia and the Tomorr populations (b = 3.3, *P* = 0.004, Fig. 2B). Latitude positively influenced *T*_*p*_ (b = 1.557, *P* = 0.006), lower set-point range of body temperature (*T*_*set*_) (b = 2.375, *P* = 0.002) and upper *T*_*set*_ (b = 0.872, *P* = 0.059; Fig. 3). The phylogenetic position in the sampled *V.* *graeca* populations did not constrain *T*_*p*_ as we found no evidence of a significant phylogenetic signal (κ = 1.13, *P* = 0.185; λ = 1.276, *P* = 0.601).

**Figure 4:**
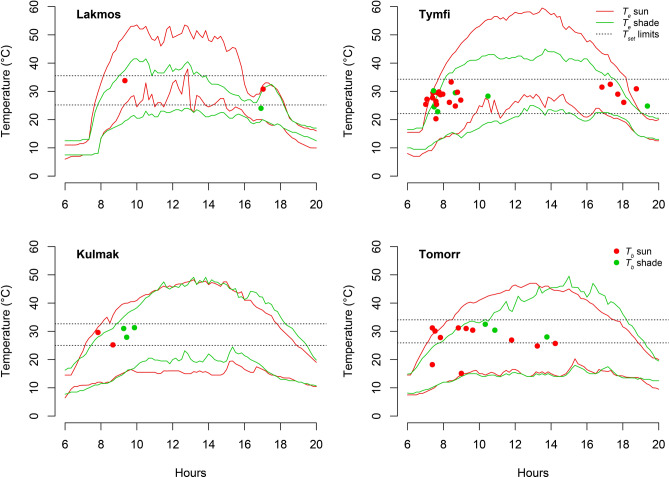
Spot-on *T*_*b*_ measurements of *Vipera* *graeca* individuals (dots) observed basking (red dots) or in shade of vegetation (green dots), and the limits of *T*_*set*_ (target body temperature, dashed lines) of each population. Solid lines indicate 95% CI range of *T*_e_ at full sun (red lines) and in the shade of vegetation (green lines) measured at four populations of *Vipera* *graeca* in July and August.

We found that the overall mean accuracy of body temperature (*d*_*b*_) was 0.45 ± 0.26 °C (mean ± SE), which indicated high accuracy of thermoregulation (Table 3). We found no difference among the populations regarding *d*_*b*_ based on the overlapping 95% CI of estimates. The mean thermal quality of the habitat (*d*_*e*_) was 4.97 ± 0.014 (mean ± SE, Table 3). At the habitats of Vardoussia and Tymfi, we found significantly lower *d*_*e*_ compared to the other habitats, indicating higher thermal quality at these sites. The cross-population mean of the effectiveness of thermoregulation sensu Hertz (*E*) was 0.92 ± 0.055 (mean ± SE, Table 3) and the effectiveness of thermoregulation sensu Boulin-Demers and Weatherhead (*I*) was 4.9 ± 0.3 (mean ± SE, Table 3). Spot-on *T*_*b*_ measurements never exceeded the upper limit of *T*_*set*_, and overall, they overlapped with the range of *T*_*set*_, except for some early morning *T*_*b*_ observations (Fig. 4). The overall exploitation of thermal landscape (*E*_*x*_) was 2.57.

**Table 3:** Thermal ecology indices of *Vipera* *graeca*.

Population	*Tp* °C	*T*_*set*_ °C	Mean *d*_*b*_ (95% CI)	Mean *d*_*e*_ (95% CI)	*E*	*I*
TO	30.09	25.91–34.06	1.42 (− 0.36–3.19)	5.89 (5.81–5.96)	0.76	4.47
KU	28.95	25.00–32.64	0.00 (0.00–0.00)	5.51 (5.44–5.58)	1.00	5.51
TY	28.74	22.13–34.31	0.07 (− 0.06–0.19)	4.42 (4.37–4.47)	0.99	4.36
LA	30.26	25.18–35.52	0.39 (− 0.38–1.17)	5.78 (5.7–5.86)	0.93	5.38
VA	26.77	21.00–32.01	–	4.42 (4.39–4.46)	–	–

In the case of VA populations, *d*_*b*_, *E* and *I* values are missing due the unavailability of *T*_*b*_ measurements.

**Figure 5:**
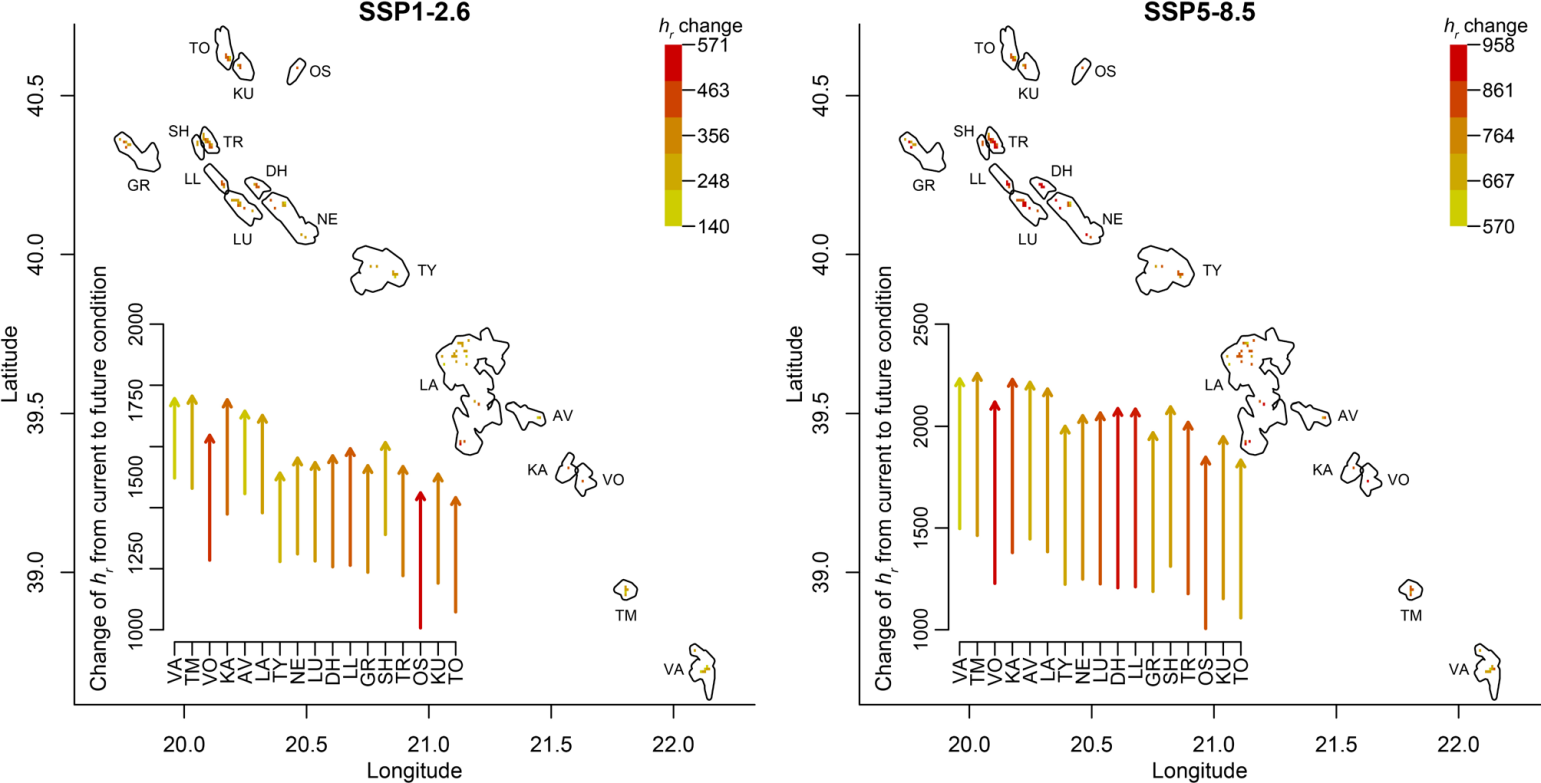
Expected change in *h*_*r*_ at *Vipera* *graeca* habitats from current climate to two future scenarios for all known localities. Inset plots show the change in mean *h*_*r*_ for each population. Colour of the arrows in the inset plot correspond to the expected change in *h*_*r*_ depicted in the maps. For populations acronyms see Fig. 1.

At current climatic conditions, the mean annual restriction time (*h*_*r*_) of *V.* *graeca* was *h*_*r*_ = 1278.4 ± 9.7 h (mean ± SE) across all localities (Fig. 5). Latitude significantly decreased *h*_*r*_ (b = − 210.9, *P* < 0.0001). For future conditions, regarding the SSP1-2.6 scenario, it is expected to increase to *h*_*r*_ = 1606 ± 9.0 h (mean ± SE), or based on the SSP5-8.5 scenario, to *h*_*r*_ = 2086 ± 8.4 h (mean ± SE). Thus, the time when *T*_*e*_ is above the upper limit of *T*_*set*_ is expected to increase by 328 or 806 h for the SSP1-2.6 and SSP5-8.5 scenarios, respectively. *h*_*r*_ was significantly higher in SSP5-8.5 compared to SSP1-2.6 (W = 2519, *P* < 0.0001).

## Discussion

Our results indicate that *T*_*p*_ of *Vipera graeca* is unaffected by sex or gravid state in females, although female size showed a positive influence on *T*_*p*_. We observed a significant difference in *T*_*p*_ only between the northernmost and the southernmost studied populations of *V.* *graeca*, however, latitude positively influenced *T*_*p*_, while we found no evidence that the phylogenetic position has a constraint. The study revealed a high accuracy of thermoregulation in *V.* *graeca* populations and some variation in the thermal quality of habitats among the studied locations. The overall effectiveness of thermoregulation was high, indicating that *V.* *graeca* is successful in achieving its target temperatures and we also found that this species effectively exploits the thermal landscape of its habitats. Under current climatic conditions, *V.* *graeca* faces a mean annual activity restriction of approximately 1278 h across all localities, and this restriction time expected to increase significantly under future climate change scenarios.

The thermal ecology of grassland vipers, including species like *Vipera* *ursinii*, *Vipera* *renardi*, and their relatives, remains largely unexplored due to the scarcity of published studies. This gap in research limits our ability to interpret our findings on *V.* *graeca* with existing literature. However, available data on *V.* *ursinii* *rakosiensis* suggest an optimal body temperature "somewhat below 35 °C", as observed in radiotagged individuals^40^. This optimal temperature is notably higher than the *T*_*p*_ and *T*_*set*_ we recorded for *V.* *graeca*. For *V.* *u.* *rakosiensis*, this temperature might align more closely with their voluntary thermal maxima (VTM) rather than their upper *T*_*set*_^37^. Further comparison with *V.* *ursinii* *moldavica*, based on spot-on *T*_*b*_ recordings, shows some overlap with our observations for *V.* *graeca*^41^. Beyond grassland vipers, additional insights can be drawn from studies on *Vipera* *berus*, which can also be categorized as a cold-adapted species. Our findings indicate that *V.* *graeca* exhibits a lower *T*_*p*_ and a much wider range of *T*_*set*_ than *V.* *berus*^14^, suggesting that *V.* *graeca* may possess a broader thermal niche.

Consistent with our findings, similar patterns have been observed in other viper species regarding the lack of significant difference in *T*_*b*_ between reproductive and non-reproductive females. Specifically, in *V.* *berus*, a study found no significant difference in *T*_*p*_ among reproductive states^42^. A parallel observation was made in *V.* *ammodytes*, where no distinct differences in *T*_*b*_ were noted concerning the reproductive state; however, gravid females tended to select warmer microhabitats, possibly to achieve more precise thermoregulation^43^. In contrast, *V.* *aspis* presents a differing scenario, where gravid females, particularly in the latter half of gestation, tend to maintain a higher *T*_*b*_^44^. This variability across species underscores the complexity of thermoregulatory behaviors in vipers and suggests potential species-specific strategies related to reproductive state and thermoregulation.

Our study revealed that *V.* *graeca* in higher latitude habitats, which are characteristically colder, exhibit a preference for higher temperatures, both in terms of *T*_*p*_ and *T*_*set*_. This finding aligns with studies indicating that reptiles in higher elevations and latitudes tend to have increased upper thermal tolerance limits, such as thermal-safety margin, critical thermal maximum (CT_max_), and VTM^11,37^. This pattern may be attributed to reptiles becoming more effective thermoregulators in challenging thermal environments with limited heat resources^45^. The absence of a significant phylogenetic influence on *T*_*p*_ in our study suggests that the observed variations in *T*_*p*_ across different populations of *V.* *graeca* could be the result of local adaptation and/or phenotypic plasticity rather than of evolutionary descent. Future research in this field should, therefore, include phenotypic plasticity and the capacity for reversible phenotypic plasticity as a fundamental component of experimental designs, providing a comprehensive understanding of how reptiles adapt to their thermal environments^46^.

Our data shows that *V.* *graeca* generally maintains its *T*_*b*_ within the preferred *T*_*set*_, except for three “cold” individuals, which were likely in the initial stages of morning basking. This accuracy of thermoregulation potentially leads individuals to maintain high physiological performance by active thermoregulation in a challenging environment. Interestingly, these snakes do not utilize the entire available thermoregulatory time window despite suitable environmental temperatures to keep *T*_*b*_ in *T*_*set*_. This behaviour might be influenced by factors beyond thermal conditions alone. For instance, a study suggest a behavioral adaptation in *V.* *graeca*, where shifts in their daily bimodal activity patterns—earlier mornings and later afternoons—could be attributed to predator avoidance strategies, particularly evasion of visually-searching raptor birds such as the short-toed snake eagle (*Circaetus* *gallicus*)^13^. Additionally, variations in the exploitation of thermally suitable times are likely to be observed across seasons, which may reflect changes in environmental conditions and the physiological needs of the individuals. Factors such as size (or age), foraging requirements, and reproductive objectives could also play significant roles in shaping these thermoregulatory activities. While most existing studies in thermal ecology, including this study, focus on short-term, intensive data collection periods, future research should aim to extend these observational windows. Covering longer periods would provide a more comprehensive understanding of how seasonal variations, life-history traits, and ecological interactions, such as predator–prey dynamics, influence the thermal ecology of *V.* *graeca* and other reptilian species.

In a previous study employing correlative species distribution modelling, we projected that around 90% of *V.* *graeca*’s current habitats might be lost by the end of the twenty-first century due to climate change^47^. These correlative models, praised for their simplicity and applicability across various species with available distribution and environmental data^48^, contrast with more accurate process-based mechanistic models^49^. The latter includes detailed physiological mechanistic models that solve coupled energy and mass balance equations to establish an explicit link between the organism’s requirements and the environmental availability of resources^50^. Our current analysis, while not forecasting changes in distribution, focuses on how thermoregulatory time budgets may shift. We observed a significant increase in *h*_*r*_, the duration when environmental temperatures surpass the upper limit of the species’ temperature preference. This rise in *h*_r_ implies reduced opportunities for thermoregulation, foraging, and reproduction, potentially leading to direct impacts on individual fitness and population growth rates. For a species such as *V.* *graeca*, which is already endangered and confined to shrinking and deteriorating mountaintop habitats, this reduction in key demographic factors could exacerbate the risk of local extinctions.

In conclusion, our study on *V.* *graeca* revealed critical insights into the species’ thermal ecology. We found that preferred body temperature varies significantly with latitude and female body size, but is not influenced by sex or reproductive state. The species shows high accuracy in thermoregulation and level of exploitation of the thermal landscape. However, our findings also indicate an impending challenge: as climate change progresses, *V.* *graeca* faces increasing restrictions in thermoregulatory opportunities, which could significantly impact its foraging and reproductive behaviours, thereby affecting overall fitness and population growth. There is a crucial need for considering both local environmental conditions and potential impacts of climate change in the conservation strategies for *V.* *graeca*, to mitigate the risks of habitat loss and population decline in this endangered species.

## Methods

### Study species

The Greek meadow viper (*Vipera* *graeca* Nilson & Andrén, 1988) is a small-sized grassland viper living exclusively in the subalpine and alpine grasslands of the Pindos mountain range in Southern Albania and Central Greece, usually between 1600 and 2100 m above sea level^47,51,52^. It has a severely fragmented distribution forming 17 known isolated populations on mountaintops above the tree line (Fig. 1)^47,53^. The habitats of the species are threatened by climate change and anthropogenic degradation such as overgrazing, and most likely 90% of the habitats will probably disappear by the end of this century^47^. The species is currently listed as endangered by the IUCN Red List^53^. The populations genetically form two major lineages, a southern (Vardoussia among the sampled populations in this study) and a northern one (all other populations sampled in this study, Fig. 1), without signs of inbreeding or genetic drift. This species is a dietary specialist on Orthopterans^54^.

### Data collection

We collected data on five populations of *Vipera* *graeca* along a latitudinal gradient from north to south: Tomorr, Kulmak (Albania), Tymfi, Lakmos and Vardoussia mountains (Greece), including the northernmost and the southernmost populations (Fig. 1). All populations were sampled in the activity peak in summer: 29 July–14 August 2017 (Tymfi), 14–22 August 2017 (Lakmos), 23 July–12 August 2018 (Vardoussia), July 25–August 15 2019 (Tomorr and Kulmak).

The evaluation of the thermoregulatory characteristics of ectotherm animals such as *V.* *graeca*, requires information on the availability and distribution of body temperatures a non-thermoregulating individual could or would achieve based purely on energy flux from radiation, conduction, and convection^16^. Accordingly, we collected three types of temperature data for this study: operative temperature (*T*_*e*_), field body temperature (*T*_*b*_) and target body temperature in a thermal gradient (i.e. set-point temperature range *T*_*set*_). Operative or environmental temperature (*T*_*e*_) is the equilibrium of *T*_*b*_, as it gives the null distribution of *T*_*b*_ expected from non-thermoregulating individuals^55^. We measured *T*_*e*_ by using the physical model (operative temperature model, OTM) made of a copper tube that mimicked the size (18 × 350 mm), shape, and heat absorption of the study species. To measure *T*_*e*_, we equipped the models with temperature data loggers (iButton DS1921G-F5#, Thermochron Ltd., Castle Hill, NSW, Australia; 0.5 °C resolution, ± 1.0 °C accuracy) pre-set to record data in 5-min intervals. We placed the models in two different micro-environments, with one exposed to the sun and the other under the shade of vegetation cover. The models were placed in the micro-environments closest to the exact location of capture of *V.* *graeca* individuals.

The distribution of body temperature of an actively thermoregulating animal is expected to differ from *T*_*e*_. To measure field *T*_*b*_ we sampled *V.* *graeca* by spot sampling often called “grab and jab” sampling^16^. We intensively searched for vipers in their habitats, and when a viper was spotted, we carefully captured it using protective gloves and immediately measured its cloaca temperature with a thermometer (Testo 103, Testo SE & Co. KGaA, Baden-Württemberg, Germany; 0.1 °C resolution, ± 0.5 °C accuracy). We recorded the GPS coordinates and the snake was subsequently transported to our field camp, where other measurements were conducted. Each individual was housed in a linen bag in a shaded area to ensure their well-being during the temporary keeping.

To better understand the thermoregulation of ectotherms it is central to identify the target body temperature that an individual would achieve^56^. Target (or preferred, or selected) body temperature can be measured in artificial thermal gradients, in an environment that is independent from the ecological costs and constraints that can influence thermoregulation in the field^16^. To measure *T*_*set*_ we kept the individuals in a thermal gradient set up in the field, from 6:00 AM to 6:00 PM and measured cloaca temperature hourly with a thermometer (Testo 826-T4, Testo SE & Co. KGaA, Baden-Württemberg, Germany; 0.1 °C resolution, ± 0.5 °C accuracy). The thermal gradient was set up in 100 × 30 × 30 cm polycarbonate terrariums (dimensions: 100 × 30 × 30 cm; n = 4) positioned under permanent shade. To establish the desired thermal gradient (max. 20 °C on the cool and min. 40 °C on the hot end), the hot side of the terrarium was heated using a 200 W ceramic heat wave source (Exo Terra PT2046; Rolf C. Hagen, Inc. Montreal, QC H9X 0A2, Canada). Additionally, to maintain an optimal temperature range, evaporative cooling was utilised whenever the air temperature at the cooler end of the gradient reached 20 °C. This was achieved by covering the initial 30 cm of the terrarium's top surface with a water-soaked textile sheet, placed at the cooler end. Thermal measurements were followed by sexing, determination of the gravidity status of females and measuring the snout-vent length (SVL) of the individuals. After measurements, snakes were released at their exact capture location.

To assess the influence of change in the thermal landscape on the activity time of vipers we compiled a database of all *V.* *graeca* occurrence records available from our previous studies and the observations recorded in this study (n = 378 records from all known populations)^37,47,51–53^. We downloaded data on monthly air temperatures at 30″ spatial resolution (~ 720 × 930 m grid) from the Worldclim 2.1. database^57^ for the entire geographic range of *V.* *graeca*. To characterize future climatic conditions (years 2081–2100), we selected three Global Climate Models (GCMs): HadGEM3-GC31-LL, IPSL-CM6A-LR, MIROC6^58^ and two Shared Socioeconomic Pathway (SSP) scenarios, an optimistic one (SSP1-2.6) and a pessimistic one (SSP5-8.5). The optimistic SSP1-2.6 scenario represents a future world characterized by low population growth, strong sustainability efforts, and significant reductions in greenhouse gas emissions, i.e., representing a future in which ambitious actions are taken to mitigate the worst effects of climate change^59^. The pessimistic SSP5-8.5 represents a high-emission scenario in which greenhouse gas concentrations continue to rise throughout the twenty-first century, leading to a world with high levels of carbon dioxide and other greenhouse gases^59^.

### Data analysis

To describe the thermoregulation of *Vipera* *graeca* we calculated the indices and variables commonly used to describe the thermoregulation of free-living animals (Table 1)^24^. All data operations and statistical analyses were conducted in the R 4.1.3 statistical environment in a fully reproducible way^60^.

Because most of the ectotherms appear to thermoregulate between an upper and lower temperature limit rather than around a single temperature value (*T*_*p*_), it is straightforward to determine *T*_*set*_. The upper and lower limits of preferred *T*_*b*_ could be estimated by calculating the central 50% of the distribution of all *T*_*b*_ values selected in a thermal gradient^16^. To calculate *T*_*p*_ and *T*_*set*_, we used an approximation function on the density distribution of body temperatures measured in thermal gradient for each individual. First, we fitted the density function to the data, second, we transformed the probability values (y-axis) to range between 0 and 1. Third, using the approxfun function we estimated the probability values for a sequence of temperature values from 15 to 45 °C by 0.05 °C steps. Finally, we read the *T*_*p*_, and *T*_*set*_ values: at probability = 1 as *T*_*p*,_ min. value at probability = 0.5 as *T*_*set*_ lower value and max. value at probability = 0.5 as *T*_*set*_ upper value.

To assess the influence of SVL on *T*_*p*_ (Q1) we fitted linear models (LM) using the lm function for female and male individuals separately. To assess the effect of gravidity on females’ *T*_*p*_ we used Wilcoxon rank test using the wilcox.test function and fitted an LM. We investigated the differences in *T*_*p*_ across populations (Q2) by fitting a LM, and then we applied multiple comparisons on the LM using the glht function of the multcomp package to identity significantly (*P* < 0.05) different groups^61^. To assess the influence of latitude on *T*_*p*_ and *T*_*set*_, we also applied LMs at the level of individuals (Q3). We fitted three LMs separately for each dependent variable (*T*_*p*_, *T*_*set*_ lower, *T*_*set*_ upper) using the same independent variable (latitude).

To test for the presence of a phylogenetic signal in *T*_*p*_ (Q3), we used the phylogenetic tree reconstruction based on RADseq data (see supplementary material). To import the tree file of that phylogenetic reconstruction and for the subsequent analysis, we applied the ape and phytools packages^62,63^. To calculate branch lengths we used the compute.brlen function of ape. We tested for the presence of phylogenetic signal by computing Pagel’s lambda^64^ and Bloomberg’s K^65^ on *T*_*p*_ using the phylosig function of phytools package.

We applied the index of accuracy of body temperature (*d*_*b*_) by calculating the mean of the deviations of *T*_*b*_ from *T*_*set*_ (Table 1)^16^. To assess the thermal quality of the habitats (*d*_*e*_), we used the mean of the deviations of *T*_*e*_ from *T*_*set*_ (Table 1)^16^. For this calculation, we used a subset of *T*_*e*_ measurements to encompass the diurnal activity time (6:00 AM to 8:00 PM) of the study species using the lubridate package^66^. *d*_*b*_ and *d*_*e*_ values with 95% confidence intervals were calculated for each measured population (n = 4 and n = 5 respectively). We assessed the effectiveness of thermoregulation (Q4) by calculating the *E* index (*E* = 1 − (*d*_*b*_/*d*_*e*_))^16^ and the *I* index (*I* = *d*_*e*_ − *d*_*b*_)^22^. To calculate the thermal exploitation of the habitats *E*_*x*_^23^, we counted cases when spot-on *T*_*b*_ measurements were inside the range of *T*_*set*_ and divided by the number of all *T*_*b*_ measurements when the corresponding *T*_*e*_ was overlapping with *T*_*set*_.

To estimate the expected change in activity time (Q5), we calculated the restriction time of activity as the number of hours during a full year (24 × 365) when *T*_*e*_ exceeded the upper limit of *T*_*set*_^24^. To get *T*_*e*_ for an entire year and all occurrence areas (n = 102 at the spatial resolution of 30″) of *V.* *graeca*, we used a microclimate model to establish a simulated environment and an ectotherm model to predict *T*_*e*_ using the NicheMapR package^67^. As the default temperature data in the micro_global modelling function has a low spatial resolution (10 × 10 km grid), we used the current (1981–2010) monthly mean air temperature data extracted for each viper occurrence grid cell from the Worldclim 2.1. database. All raster operations were done using the terra, raster and dismo packages^68–70^. We set the warm parameter to the difference of means at the fine resolution of the default monthly temperature values to establish a microclimate model for current climatic conditions. In this simulated environment, we fitted an ectotherm model by default parameters, except for the weight of the animal model (33.5 g, mean of all adult *V.* *graeca* weighted, n = 192), the shape of the animal (cylinder), and diurnality. We fitted the ectotherm model as “dead” (live parameter set to 0), meaning non-thermoregulating, as we were interested in getting *T*_*e*_ measurements. We extracted the *T*_*e*_ estimates from the ectotherm model output, and counted the number of hours when *T*_*e*_ exceeded the *T*_*set*_ (= restriction time, *h*_*r*_). To get *h*_*r*_ for future climate, we used the warm parameter similarly as above using the extracted monthly air temperature of the n = 6 future data sets (3 GCM × 2 SSP). We repeated this procedure for each location to map *h*_*r*_ for the whole distribution of *V.* *graeca*. Finally, we calculated the difference between future and climate restriction times to predict the change in *h*_*r*_ of activity, and we mapped the *h*_*r*_ results for presentation purposes to the same raster resolution as the Worldclim data we used for microclimate modelling.

### Supplementary Information

Supplementary Information.

## Data Availability

All data and code files related to this study are available at the Zenodo repository (https://doi.org/10.5281/zenodo.12771109).
