# Supplementary material for: Alpine viper in changing climate: thermal ecology and prospects of a cold-adapted reptile in the warming Mediterranean
Source: Sci Rep. 2024 Aug 16;14:18988. doi: 10.1038/s41598-024-69378-4 (PMC11329715; doi:10.1038/s41598-024-69378-4)
Supplement: Supplementary file 1 — Supplementary Information. [file 41598_2024_69378_MOESM1_ESM.docx]

**Supplementary material**

**Alpine viper in changing climate: thermal ecology and prospects of a cold-adapted reptile in the warming Mediterranean**

Edvárd Mizsei, Dávid Radovics, Gergő Rák, Mátyás Budai, Barnabás Bancsik, Márton Szabolcs, Tibor Sos, Szabolcs Lengyel

T o produce a phylogenetic tree (Figure S1.) for the relationship of studied *Vipera graeca* populations we used 210 ng of dsDNA from each sample in the RADseq library preparation following the original protocol of Baird et al. (2008) with some slight modifications outlined in Szatmári et al. (2021). Given the moderate genome size (2.18 Gbp) assumed for our focal species (https://goat.genomehubs.org/), the enzyme pstI (New England Biolabs, Ipswich, MA, USA) was used for the restriction digestion of the dsDNA. The final library was sequenced on an Illumina machine using an Illumina NextSeq 500/550 Mid Output Kit v2.5 kit (Illumina Inc., San Diego, CA, USA) with a single-end sequencing option at the Genomic Medicine and Bioinformatic Core Facility, University of Debrecen (Hungary).

Raw NextSeq reads were first demultiplexed using the process_radtags module of Stacks v.2.2 (Rochette et al., 2019). In the presence of the draft genome of a closely related species, Vipera berus (GCA_000800605.1), we applied a reference-based single nucleotide polymorphism (SNP)-calling using the ref_map module of Stacks with all default parameters. Prior to SNP calling, reads were aligned to the reference genome using bwa 0.7.17 using default options (Li, 2013). For phylogenetic analyses, all SNPs were exported into variant call format (vcf) with the population’s module of Stacks, and further filtered with vcftools v.0.1.16 (Danecek et al., 2011) requiring a locus to be present in at least 60% of the individuals, a minor allele frequency (maf) of 0.26 (i.e., all alleles are present in at least two individuals).

The dataset, containing all SNPs, we reconstructed the phylogenetic relationship between our samples using a maximum-likelihood approach as implemented in IQtree v.2.0.3 (Minh et al., 2020). The analysis was run by correcting for ascertainment bias using the automatic model selection (option ‘-m MFP+ASC’) with statistic robustness assessed via Shimodaira-Hasegawa approximate likelihood ratio test (SH-aLRT) and ultrafast bootstrap (UFBoot) with 1000 replications. Here, statistical robustness was accepted if SH-aLRT ≥ 80 and UFBoot ≥ 95.

**Figure S1.** Maximum likelihood phylogenetic reconstruction of sampled *Vipera graeca* populations based on RADseq SNP data (Mizsei et al. unpublished results.) Population ID acronyms refers to Vardoussia, Lakmos, Tymfi, Kulmak, Tomorr.


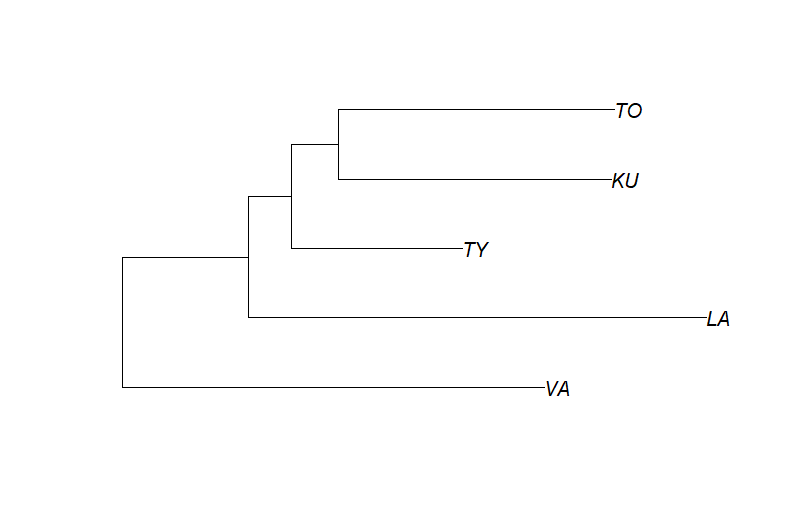
References

Baird NA, Etter PD, Atwood TS, Currey MC, Shiver AL, Lewis ZA, Selker EU, Cresko WA, Johnson EA. 2008. Rapid SNP discovery and genetic mapping using sequenced RAD markers. PLoS ONE, 3: e3376. https://doi.org/10.1371/journal.pone.0003376

Danecek P, Auton A, Abecasis G, Albers CA, Banks E, DePristo MA, Handsaker RE, Lunter G, Marth GT, Sherry ST, McVean G, Durbin R, 1000 Genomes Project Analysis Group. 2011. The variant call format and VCFtools. Bioinformatics, 27: 2156–2158. https://doi.org/10.1093/bioinformatics/btr330

Li H. 2013. Aligning sequence reads, clone sequences and assembly contigs with BWA-MEM. ArXiv, 1303.3997 [q-Bio]. http://arxiv.org/abs/1303.3997

Rochette NC, Rivera-Colón AG, Catchen JM. 2019. Stacks 2: Analytical methods for paired-end sequencing improve RADseq-based population genomics. Molecular Ecology, 28: 4737–4754. https://doi.org/10.1111/mec.15253

Minh BQ, Schmidt HA, Chernomor O, Schrempf D, Woodhams MD, von Haeseler A, Lanfear R. 2020. IQ-TREE 2: New models and efficient methods for phylogenetic inference in the genomic era. Molecular Biology and Evolution, 37(5): 1530–1534. https://doi.org/10.1093/molbev/msaa015

Szatmári L, Cserkész T, Laczkó L, Lanszki J, Pertoldi C, Abramov AV, Elmeros M, Ottlecz B, Hegyeli Z, Sramkó G. 2021. A comparison of microsatellites and genome-wide SNPs for the detection of admixture brings the first molecular evidence for hybridization between Mustela eversmanii and M. putorius (Mustelidae, Carnivora). Evolutionary Applications, 14: 2286–2304. https://doi.org/10.1111/eva.13291
